# Supplementary material for: The inhibitory mechanism of a small protein reveals its role in antimicrobial peptide sensing
Source: Proc Natl Acad Sci U S A. 2023 Oct 4;120(41):e2309607120. doi: 10.1073/pnas.2309607120 (PMC10576120; doi:10.1073/pnas.2309607120)
Supplement: Supplementary file 1 — Appendix 01 (PDF) [file pnas.2309607120.sapp.pdf]

PNAS

Supporting Information for

The inhibitory mechanism of a small protein reveals its role in antimicrobial peptide sensing

Shan Jiang, Lydia C. Steup, Charlotte Kippnich, Symela Lazaridi, Gabriele Malengo, Thomas Lemmin and Jing Yuan

Jing Yuan
Email: jing.yuan@synmikro.mpi-marburg.mpg.de

This PDF file includes:

- Supporting text
- Figures S1 to S10
- Table S1 to S3
- SI References

Other supporting materials for this manuscript include the following:

- Dataset S1

Supporting Information (SI) Text

SI Materials and Methods

Cloning and plasmid construction

E. coli phoQ-his$_6$ gene was cloned into pET Duet-1 vector at NcoI/BamHI restriction sites, and *E. coli flag-mgrB* gene was cloned into pET Duet-1 vector at NdeI/ EcoRV restriction sites. The pEVOL-pBpF vector is a gift from Dr. Hans-Georg Koch (Freiburg, Germany). Mutations in *phoQ* and *mgrB* were generated using the Q5 site-directed mutagenesis kit (New England BioLabs) following the manufacturer's instruction. All constructs were verified by DNA sequencing.

Purification of the crosslinked PhoQ-His/FLAG-MgrB complex

A two-liter culture was prepared for the purification of the crosslinked PhoQ-His/FLAG-MgrB complex. After UV crosslinking, cells were harvested, resuspended in the resuspension buffer (20 mM Tris-HCl, pH 7.5, 300 mM NaCl, 0.1 mM PMSF), and then lysed with LM10 microfluidizer at 4 °C. The lysate was centrifuged at 11,000 for 10 min to remove cell debris. The supernatant was collected and centrifuged at 13,500 g for 2 hours to pellet the total membrane, which was then dissolved in the resuspension buffer containing 1% (wt/vol) n-dodecyl-β-D-maltopyranoside (DDM) with gentle shaking at 4 °C overnight. The insoluble fraction was spun down by centrifugation at 21,000 g for 45 min. The supernatant was loaded onto a TALON column (Cytiva), which was then washed three times with buffer containing 50 mM Tris-HCl, pH 8.0, 500 mM NaCl, 10% glycerol, 10 mM imidazole, 0.1 mM PMSF and 0.03% DDM. The protein was eluted with elution buffer containing 50 mM Tris-HCl, pH 8.0, 500 mM NaCl, 10% glycerol, 200 mM imidazole, 0.1 mM PMSF, and 0.03% DDM, and then concentrated to ~200 µL. The elution was then incubated with anti-FLAG M2 magnetic beads (Sigma) at 4 °C overnight. The magnetic beads were washed with Tris-buffered saline and then incubated with 3 X FLAG peptide solution (Sigma) at room temperature for one hour to elute FLAG-tagged proteins. The supernatant was collected, concentrated to ~50 µl, and analyzed using gel electrophoresis with a 7.5% tris-glycine polyacrylamide gel.

Protein verification with mass spectrometry

Crosslinked and control bands were excised from SDS-PAGE gels and de-stained and sliced into approx. 1mm^3 pieces. Proteins in the gel pieces were reduced (10 mM dithiothreitol, 56°C for 30 min) and alkylated (50 mM chloroacetamide, 30 min, RT, in the dark) prior to overnight enzymatic hydrolysis with chymotrypsin (Promega, WI, USA) (0.1 µg of chymotrypsin per band in 50 mM ammonium bicarbonate buffer pH 7.4, 2 mM CaCl2) at 37°C. Following overnight digestion, the peptides were collected and dried down via vacuum centrifugation (Concentrator Plus, Eppendorf). Peptides were resuspended in 10 µl of HPLC loading buffer (3 % ACN + 0.1 % trifluoroacetic acid) prior to LC-MS/MS analysis.

Chromatographic separation was performed on a Dionex U3000 nanoHPLC system (Thermo, Germany) equipped with an Acclaim pepmap100 C18 column (2 µm particle size, 75 µm × 500 mm) coupled online to a QExactive Plus mass spectrometer (Thermo, Bremen). The separation was performed over a 60-minute run: 5 % B for 3 minutes, followed by linear gradients from 5 % to 50 % B over 30 minutes, then 50 % to 90 % over 1 minute, and 10 minutes at 90 % B. Inter-run equilibration was achieved by 15 minutes at 5 % B. Eluent A: 0.05 % formic acid (FA), eluent B: 80 % ACN + 0.04 % FA. A flow rate of 300 nl/min was used, and 1 µl of sample was injected per run. For crosslink samples, a second injection of 6 µl was also performed. Full scan MS acquisition was performed (300-1500 m/z, resolution 70,000, AGC target 3e6, max injection time (IT) 100 ms) with subsequent data-dependent MS/MS of the top 10 most intense ions via HCD ion activation at NCE 27 (resolution 17,500, AGC target 1e5, isolation window 1.6 m/z, max IT 50 ms); dynamic exclusion (20 s duration) and lock mass at 445.12003 m/z were enabled.

For protein identifications, the MS data files were processed with the Proteome Discoverer™ software suite (Ver. 2.5.0.400) (Thermo, Germany). Raw files were searched using the SequestHT algorithm and Target Decoy PSM validator node against a combined database that included the proteins of interest (PhoQ-Histagged, the modified MgrB-pBpA incorporation

variants), the UniProt canonical proteins for *Escherichia coli* (strain K12) (accessed: 2022.03.10), and common laboratory contaminants (cRAP list). Searches were performed with full chymotrypsin specificity and a maximum 3 missed cleavages. Precursor mass tolerance 10 ppm. Fragment mass tolerance 0.02 Da. Fixed modification of carbamidomethyl (Cys), dynamic modification oxidation (Met). Strict parsimony criteria were applied: Target FDR <1% was applied, and at least one unique high-confidence peptide was required for identification. For the small protein MgrB, a manual assessment of the peptide spectral matches was also performed.

Acceptor photobleaching FRET data acquisition and analysis
For each well, we acquired three or more sequences of images on isolated fields of view with the following protocol: (a) 2 images in the acceptor channel; (b) 140 images in the donor channel followed by (c) 16 seconds acceptor photobleaching (no image acquisition); (d) 50 images in the donor channel; and then (e) 2 images in the acceptor channel. FRET efficiency was calculated as the increase of donor fluorescence signal upon acceptor photobleaching divided by the total donor signal after acceptor photobleaching. In order to correct for the donor photobleaching present during steps b to d, we performed linear fitting (RStudio) of the donor fluorescence signal versus time for both pre- and postbleaching curves.

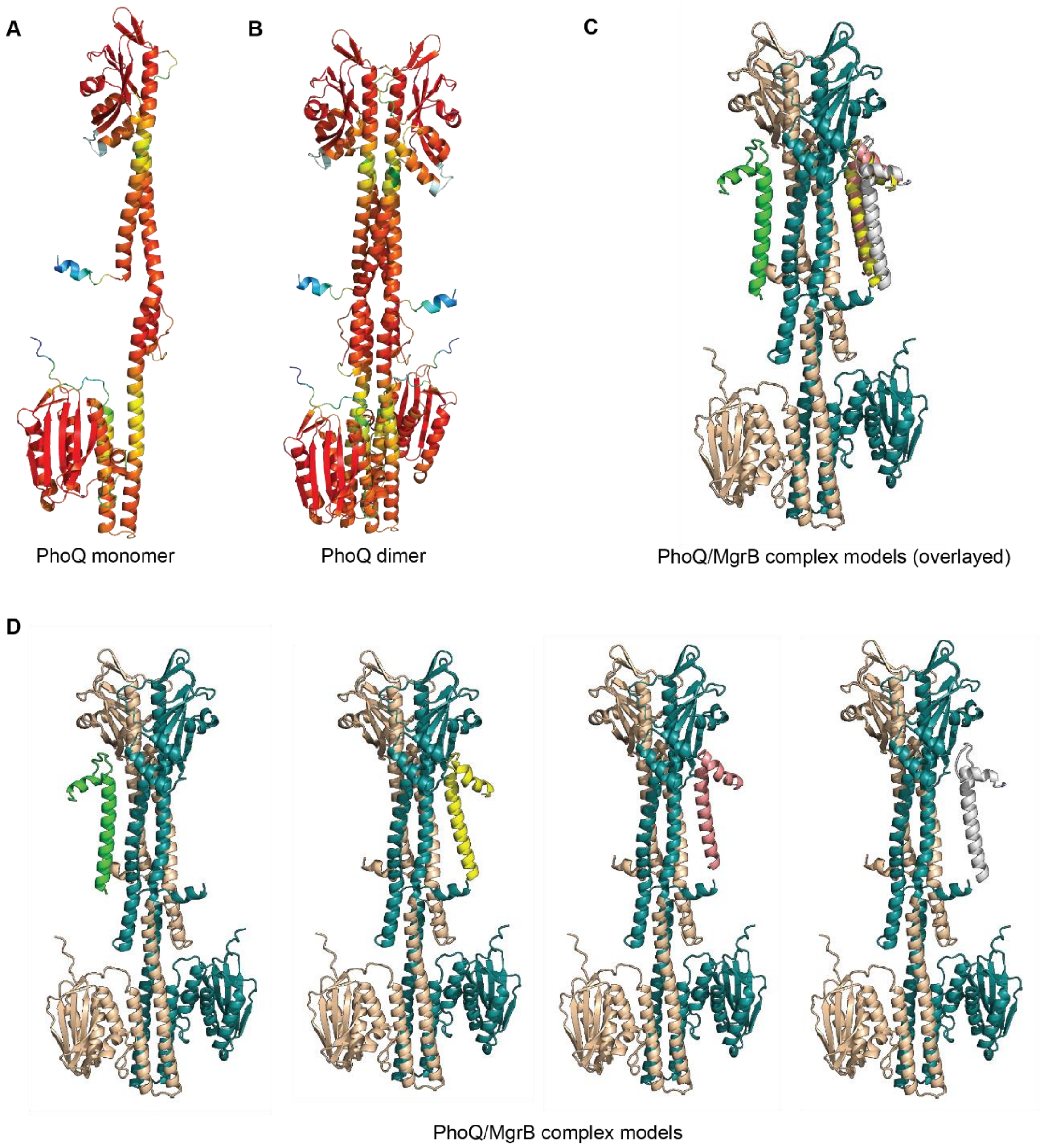

Fig. S1. Structural models predicted by Alphafold2. (**A-B**) The predicted PhoQ monomer and dimer are colored according to the pLDDT score to show the regions with lower prediction confidence. Red indicates the highest confidence and blue indicates the lowest confidence. The four predicted PhoQ/MgrB complex models ranked from two to five by Alphafold2. Models are shown with a PhoQ dimer (colored in wheat and deep teal) and four MgrB molecules overlayed (**C**) or separated (**D**) in green, yellow, pink and grey ranked from 2 to 5, respectively. All structural figures were prepared using PyMOL unless otherwise stated.

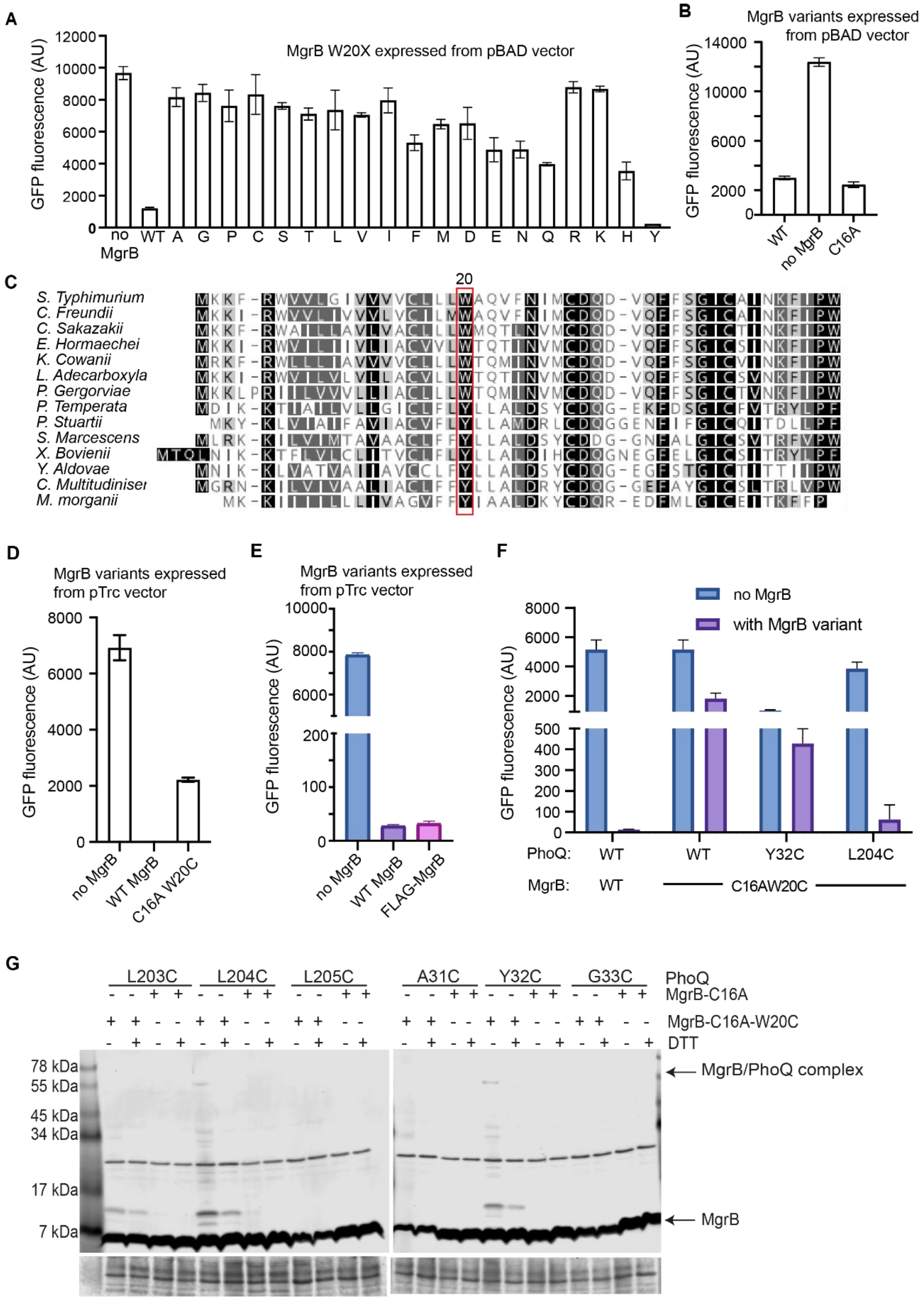

Fig. S2. The function of MgrB variants and the crosslinking with PhoQ from MgrB position 20. The activity analysis of MgrB W20 variants (**A**) and MgrB C16A (**B**) using the reporter plasmid pUA66 P_{mgtLA}-*gfp*. The *E. coli ΔmgrB* strain harboring the reporter plasmid and the pBAD plasmid

with *mgrB* variants were grown to the early log phase in LB media supplemented with 0.008% arabinose. GFP fluorescence in cells was then measured by flow cytometry. (**C**) Sequence alignment of MgrB homologs from fourteen bacterial species. The conservation of the amino acid at position 20 (*E. coli* numbering) is highlighted with a red rectangle. (**D**) The functional assay of MgrB variants expressed from pTrc99A vector. The pUA66 P_{mgtLA}-*gfp*, pBAD33 *phoQ*, and pTrc99A plasmid with flag tagged *mgrB* variants, were transformed into *E. coli ΔphoQΔmgrB* cells. The resulting transformants were grown overnight at 37 °C in LB medium supplemented with 10 mM MgSO4, then diluted 1:100 to fresh LB medium supplemented with 1 mM MgSO4, 0.008% arabinose, 10 µM IPTG, and antibiotics when appropriate. The cultures were grown at 37 °C with vigorous shaking till the early log phase. The GFP fluorescence of cells was then monitored by flow cytometry. (**E**) The functional assay of MgrB and Flag-MgrB expressed from pTrc99A vector. The pUA66 P_{mgtLA}-*gfp* and pTrc99A plasmid with *mgrB* or *flag-mgrB*, were transformed into *E. coli ΔmgrB* cells. The resulting transformants were grown overnight at 37 °C in LB medium supplemented with 10 mM MgSO4, then diluted 1:100 to fresh LB medium supplemented with 1 mM MgSO4, 10 µM IPTG, and antibiotics when appropriate. The cultures were grown at 37 °C with vigorous shaking till the early log phase. The GFP fluorescence of cells was then monitored by flow cytometry. (**F**) The functional assay of the indicated PhoQ and MgrB variants using the same protocol as in D. (**G**) Western blot analysis of *E. coli* membrane extracts after disulfide crosslinking. The indicated PhoQ and FLAG-tagged MgrB variants were expressed in *E. coli*, followed by Cu-phenanthroline catalyzed disulfide crosslinking (details in Materials and Methods). The total protein stain of the PVDF membranes serves as loading control. Data are representative of at least three independent experiments. In A, B, D, E and F, each data point is shown with the calculated average and standard deviation from three independent biological replicates.

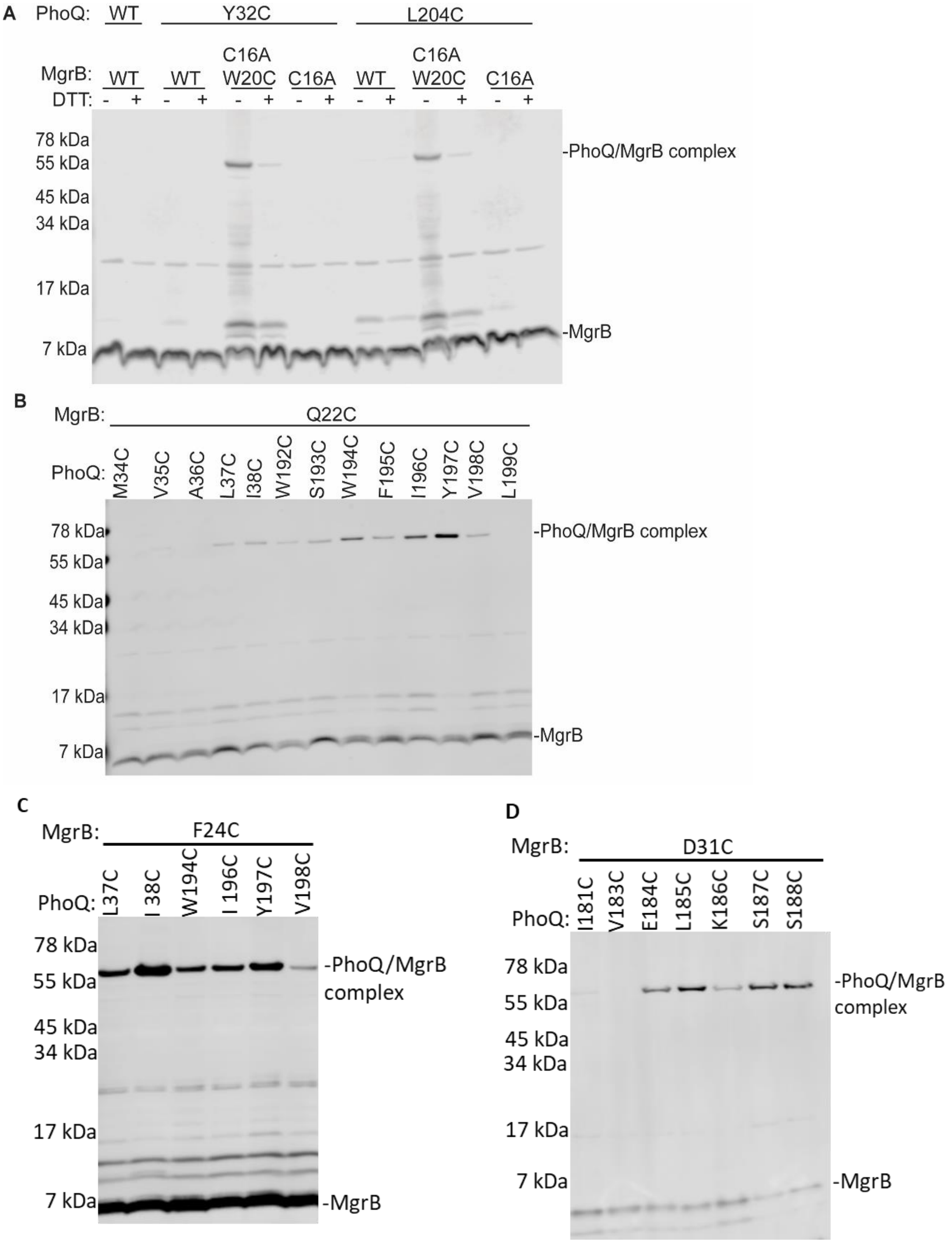
A
PhoQ: WT
Y32C
L204C
MgrB: WT
WT
C16A W20C
C16A
WT
C16A W20C
C16A
DTT: - + - + - + - + - + - + - +
78 kDa
55 kDa
45 kDa
34 kDa
17 kDa
7 kDa
-PhoQ/MgrB complex
-MgrB
B
MgrB: Q22C
PhoQ: M34C V35C A36C L37C I38C W192C S193C W194C F195C I196C Y197C V198C L199C
78 kDa
55 kDa
45 kDa
34 kDa
17 kDa
7 kDa
-PhoQ/MgrB complex
-MgrB
C
MgrB: F24C
PhoQ: L37C I 38C W194C I 196C Y197C V198C
78 kDa
55 kDa
45 kDa
34 kDa
17 kDa
7 kDa
-PhoQ/MgrB complex
-MgrB
D
MgrB: D31C
PhoQ: I181C V183C E184C L185C K186C S187C S188C
78 kDa
55 kDa
45 kDa
34 kDa
17 kDa
7 kDa
-PhoQ/MgrB complex
-MgrB

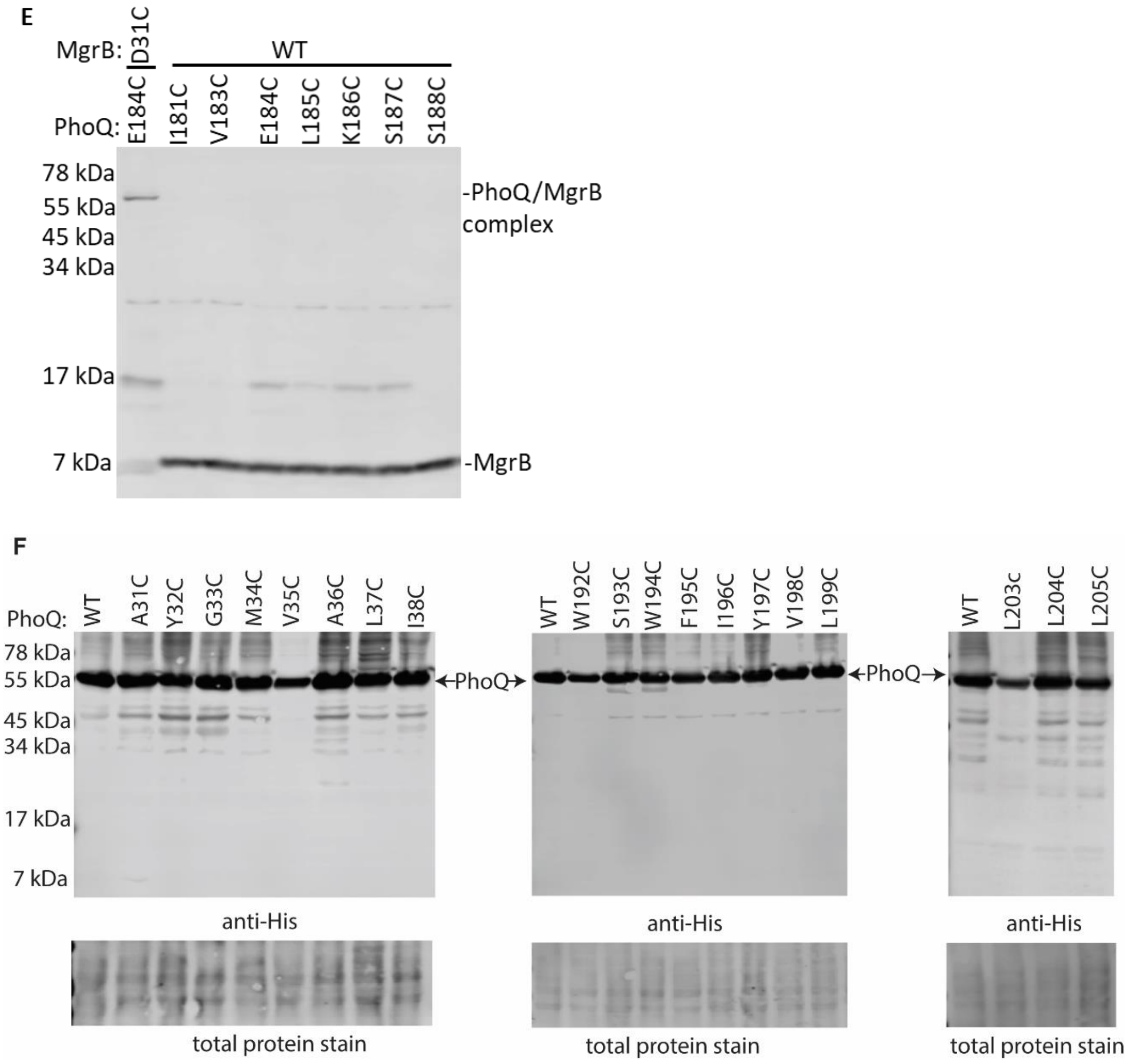

Fig. S3. The full Western blot results of the figures in the main text. (**A**) Full blot of Fig. 1D. (**B**) Full blot of Fig. 1E. (**C**) Full blot of Fig. 1F. (**D, E**) Full blots of Fig. 2B. Data are representative of at least three independent experiments. (**F**) Western blot analysis of the expression level of PhoQ variants. The indicated PhoQ-His variants were expressed in *E. coli* by inducing with 0.008% arabinose. Cells were harvested in the mid-log phase, and the total membrane was extracted for the Western blot analysis. The total protein stain of the PVDF membranes serves as a loading control. Data are representative of two independent experiments.

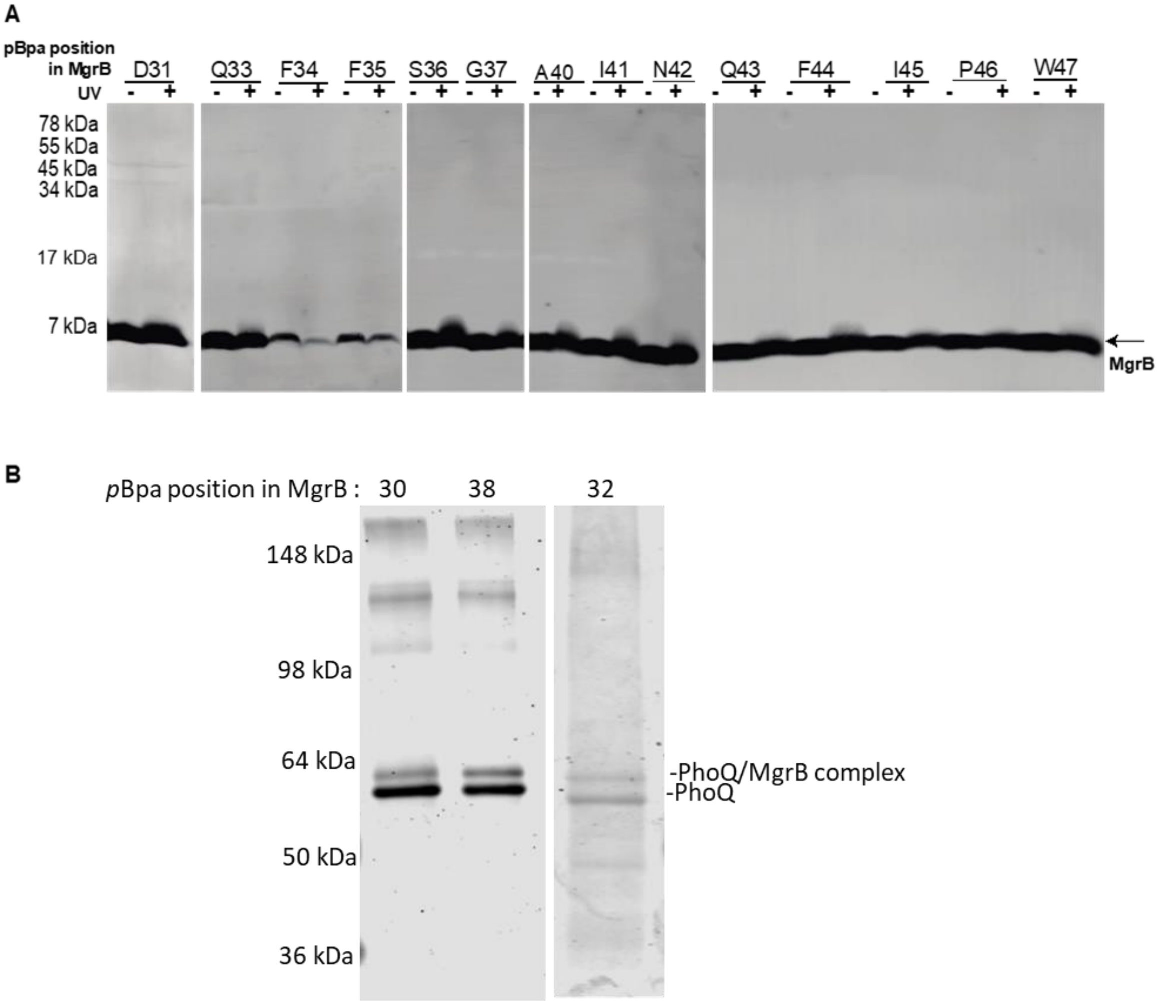

Fig. S4. Mapping the binding interface of MgrB on PhoQ by using site-specific UV crosslinking in *vivo*. (**A**) *E. coli* BL21 DE3 cells expressing PhoQ-His$_6$, FLAG-MgrB or *p*Bpa-containing FLAG-MgrB variants were irradiated with or without UV light. The cells were lysed in SDS sample buffer. Proteins were separated on 7.5% tris-glycine polyacrylamide gels and transferred to PVDF membranes. FLAG-tagged proteins were detected with an anti-FLAG primary antibody and an IRDye 800CW-conjugated secondary antibody. Data are representative of three independent experiments. (**B**) Purification of PhoQ-His/FLAG-MgrB crosslinked complex. Elution from a two-step affinity purification was separated on 7.5% tris-glycine gels. The protein bands in the gel were visualized with the ready-blue protein stain. Data are representative of two independent experiments.

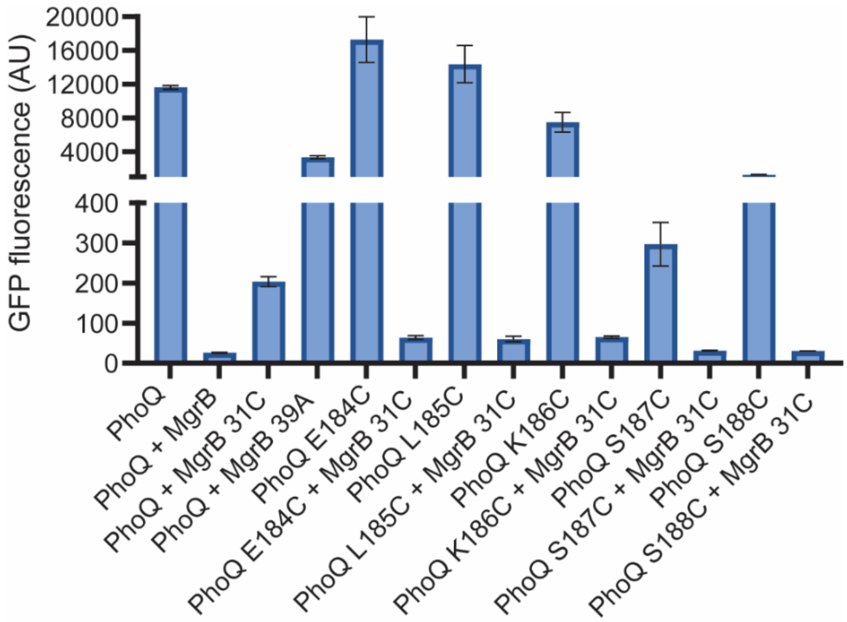

Fig. S5. The functional assay of PhoQ variants with or without MgrB. *E. coli* MG1655 *ΔphoQΔmgrB* cells harboring pBAD33 *phoQ* variants, the GFP reporter plasmid pUA66 P_{mgtLA}-*gfp*, pTrc99A with or without *mgrB* variants were grown in LB supplemented with 0.008% arabinose and 10 µM IPTG till the early-log phase. The GFP fluorescence of the cells was measured with flow cytometry. Each data point is shown with the calculated average and standard deviation from three biological replicates. The results are representative of at least three independent experiments.

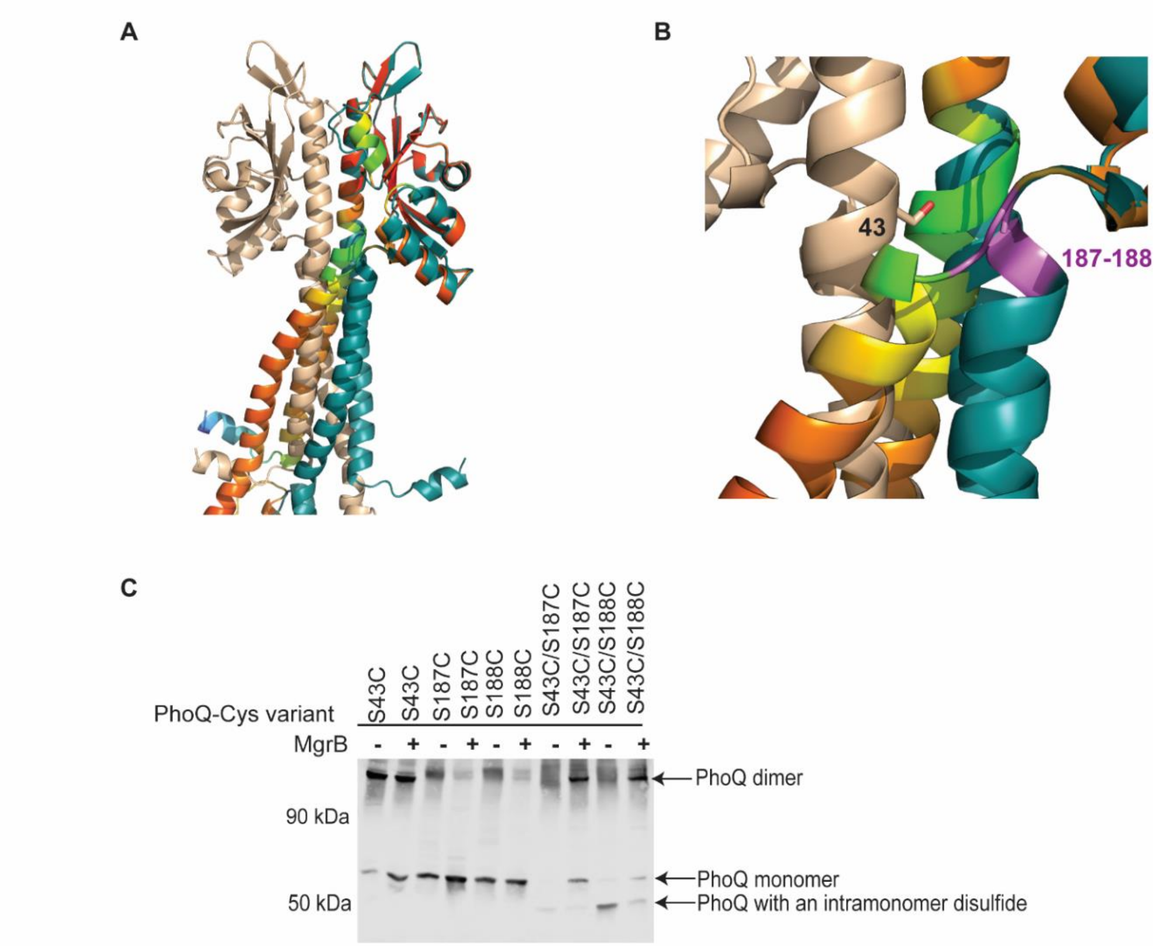

Fig. S6. The conformational change of PhoQ linker region in the presence of MgrB. (**A**) The structural superimposition of the periplasmic and TM domains of the PhoQ dimer (wheat and deep teal) and monomer (pLDDT) is shown. The periplasmic domain was used for structural alignment in Pymol. (**B**) Zoom in view of the linker region of PhoQ. Residue 43 is shown in ball-and-stick. Residues 187 and 188 are colored in purple in both monomer and dimer structures. (**C**) Western blot analysis of disulfide crosslinking within the PhoQ molecule in the presence or absence of MgrB. The crosslinked PhoQ species was detected with an anti-His antibody. Data are representative of at least three independent experiments.

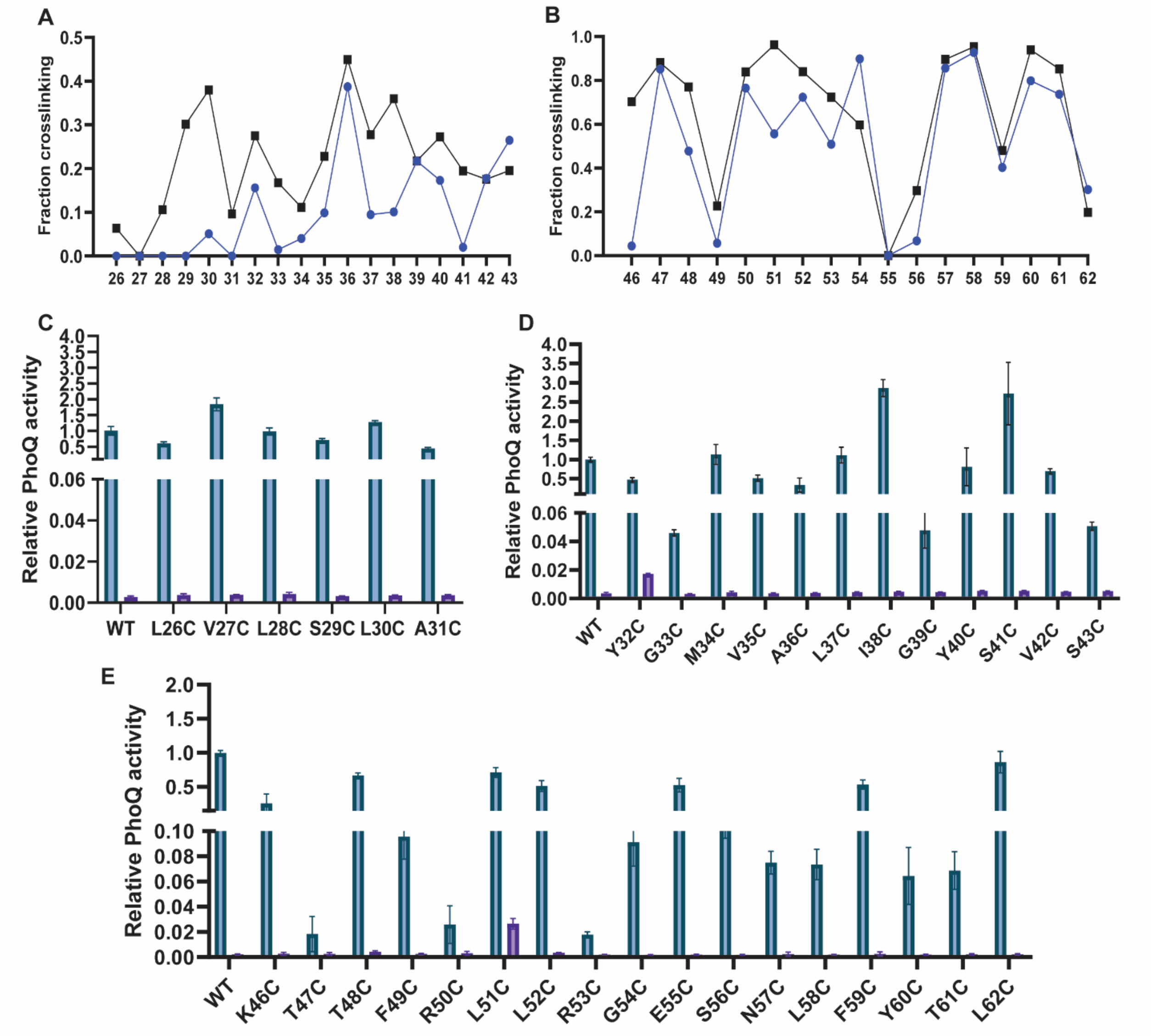

Fig. S7. **PhoQ_Cys variants in the presence or absence of MgrB.** (**A-B**) Quantification of the disulfide crosslinking efficiency of PhoQ_Cys variants in the presence (blue line) and absence (black line) of MgrB. The PhoQ dimer and monomer bands in Fig. 4C were quantified with ImageJ. The fraction of crosslinked dimer was calculated using the formula: crosslinking fraction = dimer / (dimer + monomer). (**C-E**) Functional assays of PhoQ_Cys variants with (blue) and without (purple) MgrB. *E. coli ΔphoQΔmgrB* cells harboring pUA66 P_{mgtLA}-*gfp*, pBAD33 *phoQ* variants, and pTrc99A empty or pTrc99A *mgrB* were grown overnight at 37 °C in LB medium supplemented with 10 mM $MgSO_4$. Cultures were then diluted 1:100 to fresh LB medium supplemented with 1 mM $MgSO_4$, 0.008% arabinose, and 10 µM IPTG, and grown at 37 °C with vigorous shaking until the early log phase (OD=0.4-0.5). The fluorescence of cells was monitored with flow cytometry and normalized to the PhoQ wild type. Each data point in C, D, and E is shown with the calculated average and standard deviation from three biological replicates. The results are representative of three independent experiments.

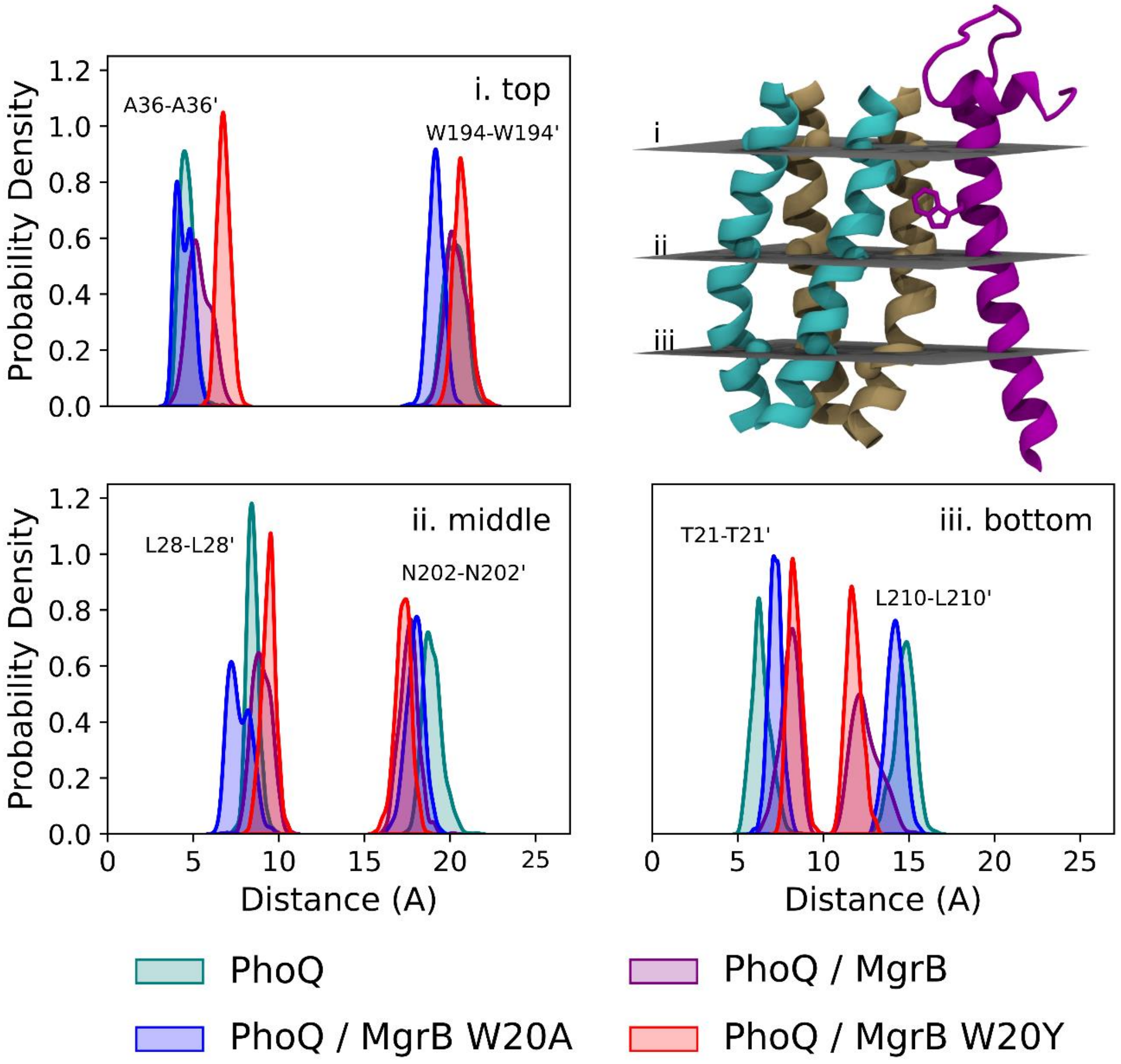

Fig. S8. Molecular dynamics simulations of PhoQ/MgrB complex models. Distribution in distances between TM1 – TM1' (left) and TM2 – TM2' (right) at the top, middle, and bottom of the PhoQ TM domain.

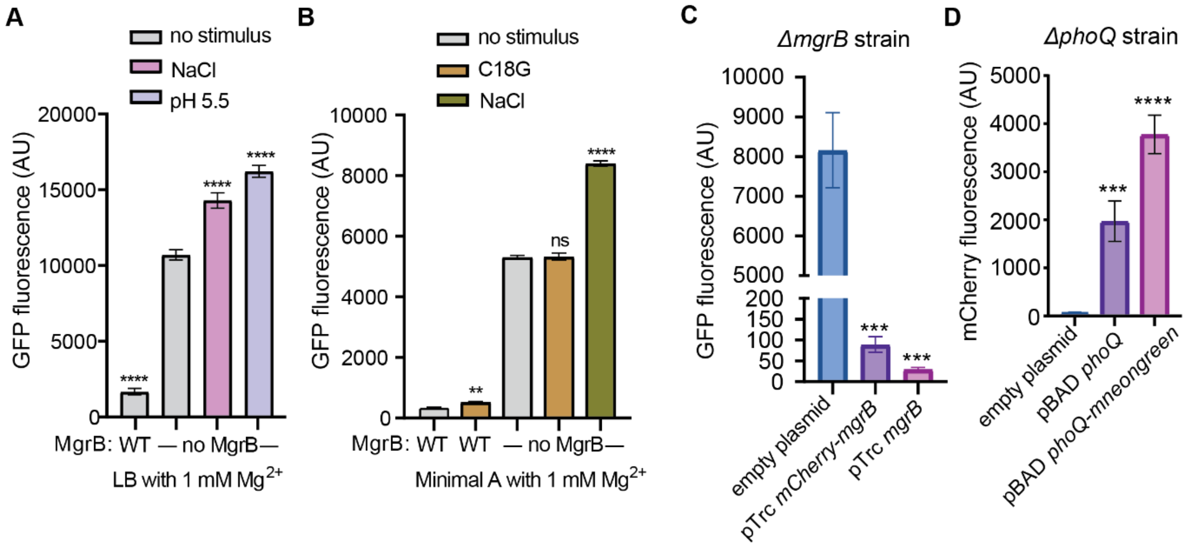

Fig. S9. The functional assays with fluorescent reporters. PhoQ activity was monitored with a GFP reporter plasmid (pUA66 P_{mgtLA}-*gfp*) with or without MgrB in LB (**A**) and in minimal A medium (**B**) supplemented with 1 mM magnesium. Briefly, *E. coli* Δ*mgrB* strain harboring the GFP reporter plasmid and a pBAD plasmid with or without the wild-type mgrB gene, was grown in the presence of the indicated PhoQ stimuli to early log phase. The GFP fluorescence of cells was then measured by flow cytometry. Data are representative of at least three independent experiments. The function of fusion proteins mCherry-MgrB (**C**) and PhoQ-mNeonGreen (**D**) was analyzed with the GFP report and a mCherry reporter (pUA66 P_{mgtLA}-*mcherry*). Indicated deletion strains were complemented with a plasmid harboring the gene of indicated fusion proteins. The expression of the fusion protein was induced with 10 µM IPTG and 0.008% arabinose for the pTrc and pBAD plasmids, respectively. Cells were grown to the early log phase and the fluorescence of cells was measured by flow cytometry. Cells transformed with empty plasmids and plasmids harboring the corresponding wild-type genes were used as negative and positive controls, respectively. Data are representative of two independent experiments.

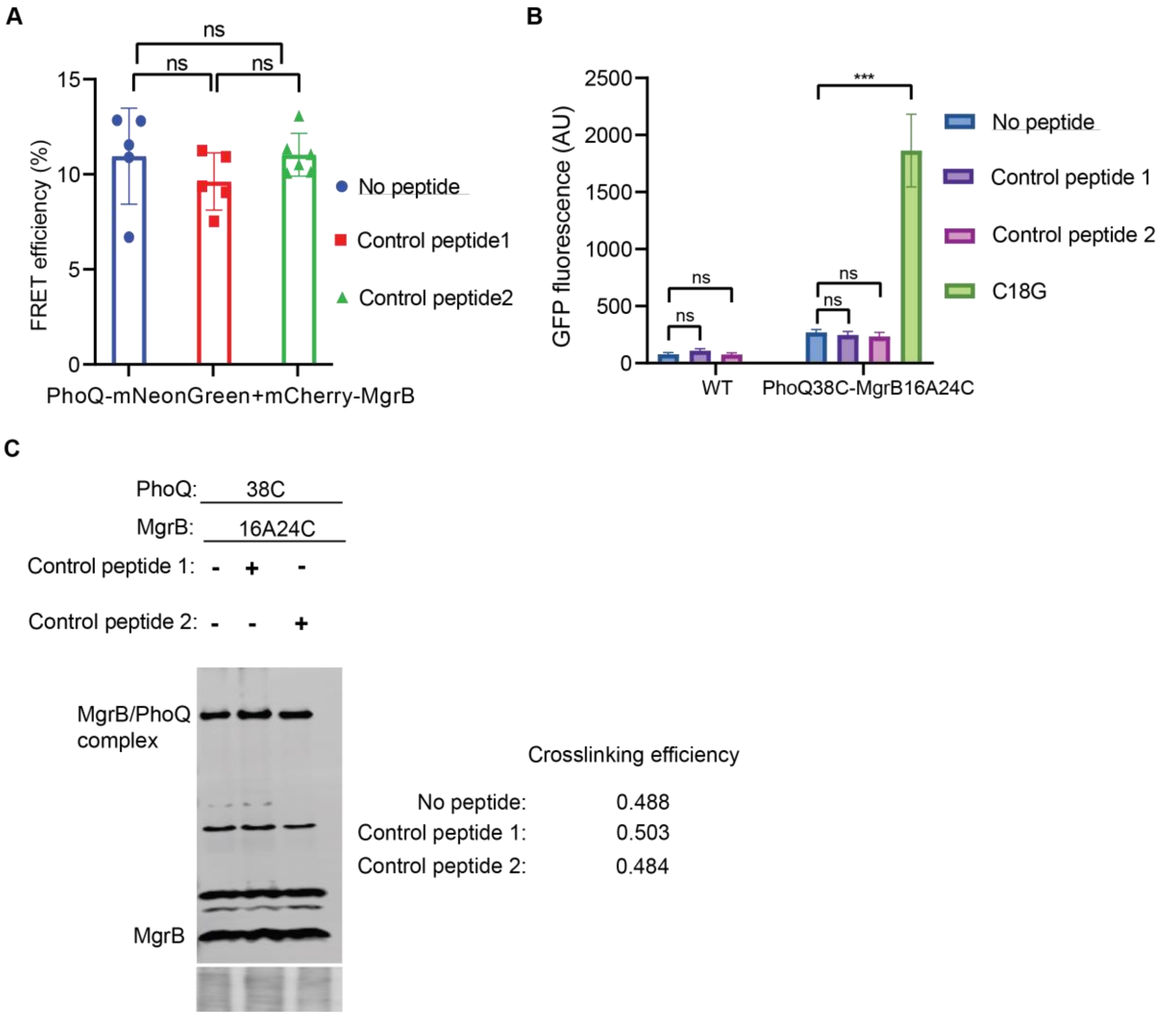

Fig. S10. Utilizing two different peptides as negative controls for FRET, GFP reporter assays, and crosslinking experiments. (**A**)The FRET measurements of PhoQ-mNeonGreen and mCherry-MgrB *in vivo* with or without two peptides (10 µg/ml) as negative controls. Peptide 1 (3 x FLAG peptide from Sigma) has a sequence of MDYKDHDGDYKDHDIDYKDDDDK. Peptide 2 has a randomly scrambled C18G sequence (KKLKKLKLKLKLSALYGALK). (**B**) The activation of PhoQ in the presence of MgrB and different peptides (5 µg/ml). (**C**) Western blot analysis of PhoQ I38C MgrB 16A24C disulfide crosslinking in the presence or absence of control peptides (5 µg/ml). The total protein stain of the membrane serves as a loading control. The crosslinking efficiency was determined by the band intensity of the PhoQ/MgrB complex vs. the sum of PhoQ/MgrB complex and MgrB monomer (analyzed using Image J). Data are representative of at least two independent experiments.

Table S1. List of strains used in this study.

Strain	Genotype	Source or Reference
JingY11	MG1655	*E. coli* Genetic Stock Center, CGSC# 7740
lptD4213	F-, *lptD4213*, *galK2*(Oc), λ^-, *IN(rrnD-rrnE)1*, *rpsL200*(strR), *rph-1*	Yale Coli Genetic Stock Center
E. coli BL21(DE3)	BL21(DE3)	New England Biolabs
JingY34	MG1655 Δ*mgrB::FRT*	[1]
JingY90	MG1655 Δ*phoQ::FRT* Δ*mgrB::FRT*	[1]

Table S2. List of plasmids used in this study.

Plasmid	Genotype	Source or Reference
pJY1	pBAD33 *phoQ-(his)*$_6$	This study
pJY31	pBAD33RBS	[2]
pJY151	pBAD33RBS *mgrB*	[1]
pOB2	pTrc99A *mcherry*	[1]
pJY569	pTrc99A *mcherry-mgrB*	[1]
pJY573	pBAD33RBS *phoQ-mneongreen*	[1]
pJY31	pUA66 EC P*mgtLA*-GFP	[2]
pJY317	pTrc99A *flag-mgrB*	This study
pJS1	pET Duet-1 EC *phoQ-his*$_6$, *flag-mgrB*	This study
pEVOL	pEVOL-pBpF	A gift from Dr. Hans-Georg Koch
pLS1	pBAD33RBS *phoQ L26C*	This study
pLS2	pBAD33RBS *phoQ V27C*	This study
pLS3	pBAD33RBS *phoQ L28C*	This study
pLS4	pBAD33RBS *phoQ S29C*	This study
pLS5	pBAD33RBS *phoQ L30C*	This study
pLS6	pBAD33RBS *phoQ A31C*	This study
pLS7	pBAD33RBS *phoQ Y32C*	This study
pLS8	pBAD33RBS *phoQ G33C*	This study
pLS9	pBAD33RBS *phoQ M34C*	This study
pLS10	pBAD33RBS *phoQ V35C*	This study
pLS11	pBAD33RBS *phoQ A36C*	This study
pLS12	pBAD33RBS *phoQ L37C*	This study

pLS13	pBAD33RBS *phoQ I38C*	This study
pLS14	pBAD33RBS *phoQ G39C*	This study
pLS15	pBAD33RBS *phoQ Y40C*	This study
pLS16	pBAD33RBS *phoQ S41C*	This study
pLS17	pBAD33RBS *phoQ V42C*	This study
pLS18	pBAD33RBS *phoQ S43C*	This study
pLS19	pBAD33RBS *phoQ F44C*	This study
pJS4	pBAD33RBS *phoQ K46C*	This study
pJS5	pBAD33RBS *phoQ T47C*	This study
pJS6	pBAD33RBS *phoQ T48C*	This study
pJS7	pBAD33RBS *phoQ F49C*	This study
pJS8	pBAD33RBS *phoQ R50C*	This study
pJS9	pBAD33RBS *phoQ L51C*	This study
pJS10	pBAD33RBS *phoQ L52C*	This study
pJS11	pBAD33RBS *phoQ R53C*	This study
pJS12	pBAD33RBS *phoQ G54C*	This study
pJS13	pBAD33RBS *phoQ E55C*	This study
pJS14	pBAD33RBS *phoQ S56C*	This study
pJS15	pBAD33RBS *phoQ N57C*	This study
pJS16	pBAD33RBS *phoQ L58C*	This study
pJS17	pBAD33RBS *phoQ F59C*	This study
pJS18	pBAD33RBS *phoQ Y60C*	This study
pJS19	pBAD33RBS *phoQ T61C*	This study
pJS20	pBAD33RBS *phoQ L62C*	This study
pJS21	pBAD33RBS *phoQ D179C*	This study

pJS22	pBAD33RBS *phoQ T181C*	This study
pJS23	pBAD33RBS *phoQ I183C*	This study
pJS24	pBAD33RBS *phoQ E184C*	This study
pJS25	pBAD33RBS *phoQ L185C*	This study
pJS26	pBAD33RBS *phoQ K186C*	This study
pLS20	pBAD33RBS *phoQ S187C*	This study
pLS21	pBAD33RBS *phoQ S188C*	This study
pLS22	pBAD33RBS *phoQ S43C-S187C*	This study
pLS23	pBAD33RBS *phoQ S43C-S188C*	This study
pCK1	pBAD33RBS *phoQ S193C*	This study
pCK2	pBAD33RBS *phoQ W194C*	This study
pCK3	pBAD33RBS *phoQ F195C*	This study
pCK4	pBAD33RBS *phoQ I196C*	This study
pCK5	pBAD33RBS *phoQ Y197C*	This study
pCK6	pBAD33RBS *phoQ V198C*	This study
pCK7	pBAD33RBS *phoQ L199C*	This study
pCK8	pBAD33RBS *phoQ S200C*	This study
pCK9	pBAD33RBS *phoQ N202C*	This study
pCK10	pBAD33RBS *phoQ L203C*	This study
pCK11	pBAD33RBS *phoQ L204C*	This study
pCK12	pBAD33RBS *phoQ L205C*	This study
pCK13	pBAD33RBS *phoQ V206C*	This study
pCK14	pBAD33RBS *phoQ I207C*	This study
pCK15	pBAD33RBS *phoQ L209C*	This study
pCK16	pBAD33RBS *phoQ L210C*	This study

pCK17	pBAD33RBS *phoQ W211C*	This study
pCK18	pBAD33RBS *phoQ V212C*	This study
pCK19	pBAD33RBS *phoQ A213C*	This study
pCK20	pTrc99A *mgrB C16A*	This study
pCK21	pTrc99A *mgrB C16A W20C*	This study
pCK22	pTrc99A *mgrB C16A Q22C*	This study
pCK23	pTrc99A *mgrB C16A F24C*	This study
pJS27	pTrc99A *mgrB D31C*	This study
pJY268	pBAD33RBS *mgrB W20A*	This study
pJY156	pBAD33RBS *mgrB C28A*	This study
pJS51	pET Duet-1 *mgrB N25-TAG*	This study
pJS52	pET Duet-1 *mgrB M26-TAG*	This study
pJS53	pET Duet-1 *mgrB M27-TAG*	This study
pJS54	pET Duet-1 *mgrB D29-TAG*	This study
pJS55	pET Duet-1 *mgrB Q30-TAG*	This study
pJS56	pET Duet-1 *mgrB D31-TAG*	This study
pJS57	pET Duet-1 *mgrB V32-TAG*	This study
pJS58	pET Duet-1 *mgrB Q33-TAG*	This study
pJS59	pET Duet-1 *mgrB F34-TAG*	This study
pJS60	pET Duet-1 *mgrB F35-TAG*	This study
pJS61	pET Duet-1 *mgrB S36-TAG*	This study
pJS62	pET Duet-1 *mgrB G37-TAG*	This study
pJS63	pET Duet-1 *mgrB I38-TAG*	This study
pJS64	pET Duet-1 *mgrB A40-TAG*	This study
pJS65	pET Duet-1 *mgrB I41-TAG*	This study

pJS66	pET Duet-1 *mgrB N42-TAG*	This study
pJS67	pET Duet-1 *mgrB Q43-TAG*	This study
pJS68	pET Duet-1 *mgrB F44-TAG*	This study
pJS69	pET Duet-1 *mgrB I45-TAG*	This study
pJS70	pET Duet-1 *mgrB P46-TAG*	This study
pJS71	pET Duet-1 *mgrB W47-TAG*	This study

Table S3. List of primers used in this study.

**Primer**	**Sequence (5' -> 3')**
pBAD33RBS *phoQ L26C* fw	AGCGGTAGTAtgcGTGCTTTCGCTTGC
pBAD33RBS *phoQ L26C* rv	GCCGTTGCCAACAGAAAA
pBAD33RBS *phoQ V27C* fw	GGTAGTACTGtgcCTTTCGCTTGCCTACGGAATG
pBAD33RBS *phoQ V27C* rv	GCTGCCGTTGCCAACAGA
pBAD33RBS *phoQ L28C* fw	AGTACTGGTGtgcTCGCTTGCCTACGGAATGG
pBAD33RBS *phoQ L28C* rv	ACCGCTGCCGTTGCCAAC
pBAD33RBS *phoQ S29C* fw	ACTGGTGCTTtgcCTTGCCTACGGAATGG
pBAD33RBS *phoQ S29C* rv	ACTACCGCTGCCGTTGCC
pBAD33RBS *phoQ L30C* fw	GGTGCTTTCGtgcGCCTACGGAATGG
pBAD33RBS *phoQ L30C* rv	AGTACTACCGCTGCCGTT
pBAD33RBS *phoQ A31C* fw	GCTTTCGCTTtgcTACGGAATGGTCG
pBAD33RBS *phoQ A31C* rv	ACCAGTACTACCGCTGCC
pBAD33RBS *phoQ Y32C* fw	TTCGCTTGCCtgcGGAATGGTCG
pBAD33RBS *phoQ Y32C* rv	AGCACCAGTACTACCGCTG
pBAD33RBS *phoQ G33C* fw	GCTTGCCTACtgcATGGTCGCGC
pBAD33RBS *phoQ G33C* rv	GAAAGCACCAGTACTACCG
pBAD33RBS *phoQ M34C* fw	TGCCTACGGAtgcGTCGCGCTGATC
pBAD33RBS *phoQ M34C* rv	AGCGAAAGCACCAGTACT
pBAD33RBS *phoQ V35C* fw	CTACGGAATGtgcGCGCTGATCG
pBAD33RBS *phoQ V35C* rv	GCAAGCGAAAGCACCAGT
pBAD33RBS *phoQ A36C* fw	CGGAATGGTCtgcCTGATCGGTTATAGCG
pBAD33RBS *phoQ A36C* rv	TAGGCAAGCGAAAGCACC
pBAD33RBS *phoQ L37C* fw	AATGGTCGCGtgcATCGGTTATAGCGTCAGTTTC
pBAD33RBS *phoQ L37C* rv	CCGTAGGCAAGCGAAAGC
pBAD33RBS *phoQ I38C* fw	GGTCGCGCTGtgcGGTTATAGCG
pBAD33RBS *phoQ I38C* rv	ATTCCGTAGGCAAGCGAAAG
pBAD33RBS *phoQ G39C* fw	CGCGCTGATCtgcTATAGCGTCAG 66C
pBAD33RBS *phoQ G39C* rv	ACCATTCCGTAGGCAAGC

pBAD33RBS *phoQ Y40C* fw	GCTGATAGGTtgcAGCGTCAGTTTC
pBAD33RBS *phoQ Y40C* rv	GCGACCATTCCGTAGGCA
pBAD33RBS *phoQ S41C* fw	GATCGGTTATtgcGTCAGTTTCGATAAAAC
pBAD33RBS *phoQ S41C* rv	AGCGCGACCATTCCGTAG
pBAD33RBS *phoQ V42C* fw	CGGTTATAGCtgcAGTTTCGATAAAACTACGTTTC
pBAD33RBS *phoQ V42C* rv	ATCAGCGCGACCATTCCG
pBAD33RBS *phoQ S43C* fw	TTATAGCGTCtgcTTCGATAAAACTACGTTTC
pBAD33RBS *phoQ S43C* rv	CCGATCAGCGCGACCATT
pBAD33RBS *phoQ F44C* fw	TAGCGTCAGTtgcGATAAAACTACG
pBAD33RBS *phoQ F44C* rv	TAACCGATCAGCGCGACC
pBAD33RBS *phoQ K46C* fw	CAGTTTCGATtgcACTACGTTTCGG
pBAD33RBS *phoQ K46C* rv	ACGCTATAACCGATCAGC
pBAD33RBS *phoQ T47C* fw	TTTCGATAAAtgcACGTTTCGGCTGTTAC
pBAD33RBS *phoQ T47C* rv	CTGACGCTATAACCGATC
pBAD33RBS *phoQ T48C* fw	CGATAAAACTtgcTTTCGGCTGTTACGTG
pBAD33RBS *phoQ T48C* rv	AAACTGACGCTATAACCG
pBAD33RBS *phoQ F49C* fw	TAAAACTACGtgcCGGCTGTTAC
pBAD33RBS *phoQ F49C* rv	TCGAAACTGACGCTATAAC
pBAD33RBS *phoQ R50C* fw	AACTACGTTTtgcCTGTTACGTG
pBAD33RBS *phoQ R50C* rv	TTATCGAAACTGACGCTATAAC
pBAD33RBS *phoQ L51C* fw	TACGTTTCGGtgcTTACGTGGCGAG
pBAD33RBS *phoQ L51C* rv	GTTTTATCGAAACTGACG
pBAD33RBS *phoQ L52C* fw	GTTTCGGCTGtgcCGTGGCGAGA
pBAD33RBS *phoQ L52C* rv	GTAGTTTTATCGAAACTGACGC
pBAD33RBS *phoQ R53C* fw	TCGGCTGTTAtgcGGCGAGAGCA
pBAD33RBS *phoQ R53C* rv	AACGTAGTTTTATCGAAACTGACG
pBAD33RBS *phoQ G54C* fw	GCTGTTACGTtgcGAGAGCAATC
pBAD33RBS *phoQ G54C* rv	CGAAACGTAGTTTTATCGAAAC
pBAD33RBS *phoQ E55C* fw	GTTACGTGGCtgcAGCAATCTGTTC
pBAD33RBS *phoQ E55C* rv	AGCCGAAACGTAGTTTTATC

pBAD33RBS *phoQ S56C* fw	ACGTGGCGAGtgcAATCTGTTCT
pBAD33RBS *phoQ S56C* rv	AACAGCCGAAACGTAGTTTTATC
pBAD33RBS *phoQ N57C* fw	TGGCGAGAGCtgcCTGTTCTATACC
pBAD33RBS *phoQ N57C* rv	CGTAACAGCCGAAACGTA
pBAD33RBS *phoQ L58C* fw	CGAGAGCAATtgcTTCTATACCCTTGCGAAGTG
pBAD33RBS *phoQ L58C* rv	CCACGTAACAGCCGAAAC
pBAD33RBS *phoQ F59C* fw	GAGCAATCTGtgcTATACCCTTGCG
pBAD33RBS *phoQ F59C* rv	TCGCCACGTAACAGCCGA
pBAD33RBS *phoQ Y60C* fw	CAATCTGTTCtgcACCCTTGCGAAGTG
pBAD33RBS *phoQ Y60C* rv	CTCTCGCCACGTAACAGC
pBAD33RBS *phoQ T61C* fw	TCTGTTCTATtgcCTTGCGAAGTGGG
pBAD33RBS *phoQ T61C* rv	TTGCTCTCGCCACGTAAC
pBAD33RBS *phoQ L62C* fw	GTTCTATACCtgcGCGAAGTGGGAAAACAATAAG
pBAD33RBS *phoQ L62C* rv	AGATTGCTCTCGCCACGT
pBAD33RBS *phoQ D179C* fw	TGTGGTGGTGtgcACCATTCCGG
pBAD33RBS *phoQ D179C* rv	ATGGTTAATTTTGGCATCC
pBAD33RBS *phoQ T181C* fw	GGTGGATACCtgcCCGGTGGAGC
pBAD33RBS *phoQ T181C* rv	ACCACAATGGTTAATTTTGG
pBAD33RBS *phoQ I183C* fw	TACCATTCCGtgcGAGCTAAAAAGTTCCTATATGG
pBAD33RBS *phoQ I183C* rv	TCCACCACCACAATGGTT
pBAD33RBS *phoQ E184C* fw	CATTCCGGTGtgcCTAAAAAGTTCCTATATG
pBAD33RBS *phoQ E184C* rv	GTATCCACCACCACAATG
pBAD33RBS *phoQ L185C* fw	TCCGGTGGAGtgcAAAAGTTCCTATATG
pBAD33RBS *phoQ L185C* rv	ATGGTATCCACCACCACA
pBAD33RBS *phoQ K186C* fw	GGTGGAGCTAtgcAGTTCCTATATGGTC
pBAD33RBS *phoQ K186C* rv	GGAATGGTATCCACCACC
pBAD33RBS *phoQ S187C* fw	GGAGCTAAAAtgcTCCTATATGGTCTG
pBAD33RBS *phoQ S187C* rv	ACCGGAATGGTATCCACC
pBAD33RBS *phoQ S188C* fw	GCTAAAAAGTtgcTATATGGTCTGG
pBAD33RBS *phoQ S188C* rv	TCCACCGGAATGGTATCC

pBAD33RBS *phoQ S193C* fw	TATGGTCTGGtgcTGGTTTATCTATG
pBAD33RBS *phoQ S193C* rv	TAGGAACTTTTTAGCTCCAC
pBAD33RBS *phoQ W194C* fw	GGTCTGGAGCtgcTTTATCTATG
pBAD33RBS *phoQ W194C* rv	ATATAGGAACTTTTTAGCTCC
pBAD33RBS *phoQ F195C* fw	CTGGAGCTGGtgcATCTATGTGC
pBAD33RBS *phoQ F195C* rv	ACCATATAGGAACTTTTTAGC
pBAD33RBS *phoQ I196C* fw	GAGCTGGTTTtgcTATGTGCTCTC
pBAD33RBS *phoQ I196C* rv	CAGACCATATAGGAACTTTTTAG
pBAD33RBS *phoQ Y197C* fw	CTGGTTTATCtgcGTGCTCTCAG
pBAD33RBS *phoQ Y197C* rv	CTCCAGACCATATAGGAAC
pBAD33RBS *phoQ V198C* fw	GTTTATCTATtgcCTCTCAGCCAATCTGC
pBAD33RBS *phoQ V198C* rv	CAGCTCCAGACCATATAG
pBAD33RBS *phoQ L199C* fw	TATCTATGTGtgcTCAGCCAATCTGC
pBAD33RBS *phoQ L199C* rv	AACCAGCTCCAGACCATA
pBAD33RBS *phoQ S200C* fw	CTATGTGCTCtgcGCCAATCTGC
pBAD33RBS *phoQ S200C* rv	ATAAACCAGCTCCAGACC
pBAD33RBS *phoQ N202C* fw	GCTCTCAGCCtgcCTGCTGTTAG
pBAD33RBS *phoQ N202C* rv	ACATAGATAAACCAGCTC
pBAD33RBS *phoQ L203C* fw	CTCAGCCAATtgcCTGTTAGTGATCCC
pBAD33RBS *phoQ L203C* rv	AGCACATAGATAAACCAG
pBAD33RBS *phoQ L204C* fw	AGCCAATCTGtgcTTAGTGATCCCGC
pBAD33RBS *phoQ L204C* rv	GAGAGCACATAGATAAACC
pBAD33RBS *phoQ L205C* fw	CAATCTGCTGtgcGTGATCCCGC
pBAD33RBS *phoQ L205C* rv	GCTGAGAGCACATAGATAAAC
pBAD33RBS *phoQ V206C* fw	TCTGCTGTTAtgcATCCCGCTGCTGTG
pBAD33RBS *phoQ V206C* rv	TTGGCTGAGAGCACATAG
pBAD33RBS *phoQ I207C* fw	GCTGTTAGTGtgcCCGCTGCTGT
pBAD33RBS *phoQ I207C* rv	AGATTGGCTGAGAGCACATAG
pBAD33RBS *phoQ L209C* fw	AGTGATCCCGtgcCTGTGGGTCG
pBAD33RBS *phoQ L209C* rv	AACAGCAGATTGGCTGAG

pBAD33RBS *phoQ L210C* fw	GATCCCGCTGtgcTGGGTCGCCG
pBAD33RBS *phoQ L210C* rv	ACTAACAGCAGATTGGCTG
pBAD33RBS *phoQ W211C* fw	CCCGCTGCTGtgcGTCGCCGCCT
pBAD33RBS *phoQ W211C* rv	ATCACTAACAGCAGATTGGCTGAGAGCACATAG
pBAD33RBS *phoQ V212C* fw	GCTGCTGTGGtgcGCCGCCTGGT
pBAD33RBS *phoQ V212C* rv	GGGATCACTAACAGCAGATTGGC
pBAD33RBS *phoQ A213C* fw	GCTGTGGGTCtgcGCCTGGTGGA
pBAD33RBS *phoQ A213C* rv	AGCGGGATCACTAACAGC
pTrc99A *mgrB C16A*	GGTGTTGGCTgcgTTGCTGCTTTGGG
pTrc99A *mgrB C16A*	ACGACAACCAGAACGACC
pTrc99A *mgrB C16A W20C* fw	GTTGCTGCTTtgcGCGCAGGTATTC
pTrc99A *mgrB C16A W20C* rv	GCAGCCAACACCACGACA
pTrc99A *mgrB C16A Q22C* fw	GCTTTGGGCGtgcGTATTCAACATGATGTGCG
pTrc99A *mgrB C16A Q22C* rv	AGCAACGCAGCCAACACC
pTrc99A *mgrB C16A F24C* fw	GGCGCAGGTAtgcAACATGATGTG
pTrc99A *mgrB C16A F24C* rv	CAAAGCAGCAACGCAGCC
pTrc99A *mgrB D31C*	GTGCGATCAGtgcGTACAATTTTTCAGC
pTrc99A *mgrB D31C*	ATCATGTTGAATACCTGC
pBAD33RBS *mgrB W20A*	CTTGCTGCTTgcgGCGCAGGTATTC
pBAD33RBS *mgrB W20A*	CAAGCCAACACCACGACA
pBAD33RBS *mgrB C28A*	CAACATGATGgcgGATCAGGATGTACAATTTTTCAGC
pBAD33RBS *mgrB C28A*	AATACCTGCGCCCAAAGC
pET Duet-1 *mgrB N25-TAG* fw	GCAGGTATTCtagATGATGTGCGATCAG
pET Duet-1 *mgrB N25-TAG* rv	GCCCAAAGCAGCAAGCAA
pET Duet-1 *mgrB M26-TAG* fw	GGTATTCAACtagATGTGCGATCAGGATGTACAATTTTTC
pET Duet-1 *mgrB M26-TAG* rv	TGCGCCCAAAGCAGCAAG
pET Duet-1 *mgrB M27-TAG* fw	ATTCAACATGtagTGCGATCAGGATGTACAATTTTTCAGCG
pET Duet-1 *mgrB M27-TAG* rv	ACCTGCGCCCAAAGCAGC
pET Duet-1 *mgrB D29-TAG* fw	CATGATGTGCtagCAGGATGTACAATTTTTC
pET Duet-1 *mgrB D29-TAG* rv	TTGAATACCTGCGCCCAA

pET Duet-1 *mgrB Q30-TAG* fw	GATGTGCGATtagGATGTACAATTTTTC
pET Duet-1 *mgrB Q30-TAG* rv	ATGTTGAATACCTGCGCC
pET Duet-1 *mgrB D31-TAG* fw	GTGCGATCAGtagGTACAATTTTTC
pET Duet-1 *mgrB D31-TAG* rv	ATCATGTTGAATACCTGC
pET Duet-1 *mgrB V32-TAG* fw	CGATCAGGATtagCAATTTTTCAGCGGAATTTG
pET Duet-1 *mgrB V32-TAG* rv	CACATCATGTTGAATACCTG
pET Duet-1 *mgrB Q33-TAG* fw	TCAGGATGTAtagTTTTTCAGCG
pET Duet-1 *mgrB Q33-TAG* rv	TCGCACATCATGTTGAATAC
pET Duet-1 *mgrB F34-TAG* fw	GGATGTACAAtagTTCAGCGGAATTTG
pET Duet-1 *mgrB F34-TAG* rv	TGATCGCACATCATGTTG
pET Duet-1 *mgrB F35-TAG* fw	TGTACAATTTtagAGCGGAATTTGTG
pET Duet-1 *mgrB F35-TAG* rv	TCCTGATCGCACATCATG
pET Duet-1 *mgrB S36-TAG* fw	ACAATTTTTCtagGGAATTTGTGCCATTAACCAG
pET Duet-1 *mgrB S36-TAG* rv	ACATCCTGATCGCACATC
pET Duet-1 *mgrB G37-TAG* fw	ATTTTTCAGCtagATTTGTGCCATTAACC
pET Duet-1 *mgrB G37-TAG* rv	TGTACATCCTGATCGCAC
pET Duet-1 *mgrB I38-TAG* fw	TTTCAGCGGAtagTGTGCCATTAACC
pET Duet-1 *mgrB I38-TAG* rv	AATTGTACATCCTGATCG
pET Duet-1 *mgrB A40-TAG* fw	CGGAATTTGTtagATTAACCAGTTTATC
pET Duet-1 *mgrB A40-TAG* rv	CTGAAAAATTGTACATCCTG
pET Duet-1 *mgrB I41-TAG* fw	AATTTGTGCCtagAACCAGTTTATCC
pET Duet-1 *mgrB I41-TAG* rv	CCGCTGAAAAATTGTACATC
pET Duet-1 *mgrB N42-TAG* fw	TTGTGCCATTtagCAGTTTATCC
pET Duet-1 *mgrB N42-TAG* rv	ATTCCGCTGAAAAATTGTAC
pET Duet-1 *mgrB Q43-TAG* fw	TGCCATTAACtagTTTATCCCGTG
pET Duet-1 *mgrB Q43-TAG* rv	CAAATTCCGCTGAAAAATTGTAC
pET Duet-1 *mgrB F44-TAG* fw	CATTAACCAGtagATCCCGTGGTG
pET Duet-1 *mgrB F44-TAG* rv	GCACAAATTCCGCTGAAAAATTG
pET Duet-1 *mgrB I45-TAG* fw	TAACCAGTTTtagCCGTGGTGACTG
pET Duet-1 *mgrB I45-TAG* rv	ATGGCACAAATTCCGCTG

pET Duet-1 *mgrB P46-TAG* fw	CCAGTTTATCtagTGGTGACTGC
pET Duet-1 *mgrB P46-TAG* rv	TTAATGGCACAAATTCCG
pET Duet-1 *mgrB W47-TAG* fw	GTTTATCCCGtagTGACTGCAGG
pET Duet-1 *mgrB W47-TAG* rv	TGGTTAATGGCACAAATTCC
Nde1 *flag-mgrB* fw	aaaCATATGGATTATAAAGATGATGATGATAAAGGTATGGTGAGCAAGGGCGAGGAG
EcoRV *flag-mgrB* rv	aaaGATATCTCACCACGGGATAAACTG
Nco1 *phoQ(his)$_6$* fw	aaaCCATGGGCAAAAAATTACTGCGTCTTTTTTTCCCGC
BamH1 *phoQ(his)$_6$* rv	aaaGGATCCTTAATGGTGATGGTGATGGTGGC

Dataset S1 (separate file). Mass spectrometry (MS) analysis of site-specific photo-crosslinking experiments.

SI References

1. S. S. Yadavalli, et al., Functional determinants of a small protein controlling a broadly conserved bacterial sensor kinase. *J Bacteriol*, **202**, e00305-20 (2020).
2. J. Yuan, et al., Osmosensing by the bacterial PhoQ/PhoP two-component system. *Proc Natl Acad Sci U S A*, **114**(50), E10792-E10798 (2017).
